# Plasma S100A8 and S100A9 Are Strong Prognostic Factors for Hepatitis B Virus-Related Acute-on-Chronic Liver Failure

**DOI:** 10.1155/2023/6164611

**Published:** 2023-07-10

**Authors:** Yao Zhang, Xueyun Zhang, Jiajia Han, Yifei Guo, Jingjing He, Feifei Yang, Richeng Mao, Yuxian Huang, Jiming Zhang

**Affiliations:** ^1^Department of Infectious Diseases, Shanghai Key Laboratory of Infectious Diseases and Biosafety Emergency Response, Shanghai Institute of Infectious Diseases and Biosecurity, National Medical Center for Infectious Diseases, Huashan Hospital, Fudan University, Shanghai, China; ^2^Key Laboratory of Medical Molecular Virology (MOE/NHC/CAMS), Shanghai Frontiers Science Center of Pathogenic Microorganisms and Infection, School of Basic Medical Sciences, Shanghai Medical College, Fudan University, Shanghai, China; ^3^Department of Hepatitis Disease, Shanghai Public Health Clinical Center, Fudan University, Shanghai, China

## Abstract

**Objectives:**

The rapidly evolving organ failure and high short-run mortality of acute-on-chronic liver failure (ACLF) are inseparable from the role of systemic inflammatory response. S100A8 and S100A9 are associated with the excessive cytokine storm and play a decisive part within the process of inflammation. We aimed to clarify the role of them in predicting prognosis of hepatitis B virus-related ACLF (HBV-ACLF).

**Methods:**

S100A8 and S100A9 levels were analyzed in plasma of 187 transplant-free HBV-ACLF patients, 28 healthy controls and 40 chronic hepatitis B (CHB) patients. S100A8 and S100A9 mRNAs were checked in liver samples from 32 HBV-ACLF patients with liver transplantation, 19 patients undergoing surgery for hepatic hemangioma and 10 CHB patients with needle biopsy.

**Results:**

The plasma levels of the S100A8 and S100A9 were higher in HBV-ACLF patients than in CHB patients (S100A8 : *P* < 0.001 and S100A9 : *P* < 0.001) and healthy controls (S100A8 : *P* < 0.001 and S100A9 : *P* < 0.001), and similar results were obtained for mRNA expression. Moreover, both proteins were related to ACLF grade, different types of organ failure, and infection, and they correlated with other prognostic scoring systems. S100A8 and S100A9 can dependently predict 28/90-day mortality (28-day: S100A8: hazard ratio (HR): 1.027; 95% confidence interval (CI): 1.007–1.048; *P*=0.026, S100A9 : HR: 1.009; 95% CI: 1.001–1.017; *P*=0.007, 90-day: S100A8 : HR: 1.023; 95% CI: 1.011–1.035; *P*=0.004, S100A9 : HR: 1.008; 95% CI: 1.004–1.012; and *P* < 0.001). Among all of the scoring systems, the combined scoring model (S100A8 and S100A9 jointly with the Chronic Liver Failure-Consortium Organ Failure score (CLIF-C OFs)) displayed the highest area under the receiver operating curve (0.923 (95% CI, 0.887–0.961)) in the prediction of 90-day mortality.

**Conclusions:**

S100A8 and S100A9 are promising biomarkers for the analysis of risk stratification and prognosis in ACLF patients. In addition, combining them with the CLIF-C OFs may better predict the prognosis of ACLF.

## 1. Introduction

Acute-on-chronic liver failure (ACLF) has been thought-about to be associate acute deterioration of liver function in patients with chronic liver diseases and is related to a high short-run fatality rate [1–3]. In Europe, ACLF is most often caused by hepatitis C or alcohol, while in Asia Pacific and Africa, ACLF is usually caused by hepatitis B virus (HBV) infection and in excess of 70% of ACLF cases are HBV-related [4, 5]. Until now, it has not been fully clarified about the pathogenesis of HBV-ACLF, but growing evidence indicates that sustained systemic inflammatory response induced by cytokine storm is crucial for the occurrence of multiple organ failure and high mortality [6, 7].

As members of S100 family, S100A8, and S100A9 are in the main derived from immunocytes, such as macrophages and neutrophils. They have already been linked to the excessive cytokine storm and make a contribution to the development of inflammation [8, 9]. The release of S100A8 and S100A9 can adjust leukocyte adhesion and slow rolling and induce secretion of multiple cytokines as a result of sustain and exacerbate inflammation [10]. Previous studies have reported that S100A8 and S100A9 have potential as reliable diagnostic and predictive biomarkers [11, 12]. Many research studies have found that S100A8 and S100A9 show important advantages over conventional biomarkers in rheumatism, systemic lupus erythematosus, inflammatory bowel disease, and other diseases [8, 12–15]. Of note, systemic inflammation triggered by exogenous or endogenous inducers is a hallmark of ACLF, and as alarmins of inflammation, the significant increase of S100A8 and S100A9 occurs in almost all kinds of inflammation. Nevertheless, the role of S100A8 and S100A9 in HBV-ACLF are poorly understood. Considering that HBV-ACLF progresses rapidly and has high mortality, it is vital to identify markers which will facilitate to predict prognosis of the disease and support appropriate treatment decision.

Hence, we explored S100A8 and S100A9 levels in HBV-ACLF patients, determined the association between these two markers and prognosis in ACLF; in addition, we also explored the potential of these two proteins as evaluation of prognosis targets in HBV-ACLF.

## 2. Materials and Methods

### 2.1. Patients

From March 2017 to June 2021, 219 patients (32 of them underwent liver transplantation among 28 days) who met the Asian Pacific Association for the Study of the Liver (APASL) HBV-ACLF criteria were enrolled [16]. In the subsequent treatment process, the patients were hospitalized at the Shanghai Public Health Clinical Center or Huashan Hospital, Fudan University. Healthy controls (*n* = 28) and chronic hepatitis B (CHB) patients (*n* = 40) were also included, and no important distinction in age and gender were ascertained among groups. CHB was defined as hepatitis B surface antigen seropositive status beyond 6 months [17]. Moreover, 19 patients with hepatic hemangioma were included. The exclusion criteria were as follows: (i) under 18 or over 80 years old, (ii) pregnancy, (iii) coinfection with human immunodeficiency virus or hepatitis C, (iv) presence of serious diseases that may have an impact on research results, including clinically significant and poorly controlled pulmonary, renal, cardiac, digestive, vascular, metabolic diseases, or cancer, and (v) loss to follow-up within 90 days.

We have obtained written informed consent from patient's legal representative or the patient himself before their enrollment. The study was performed in conformity to the tenets of the Declaration of Helsinki and was approved by the Ethical Committee of the Shanghai Public Health Clinical Center and the Huashan Hospital of Fudan University.

### 2.2. Clinical Samples and Information Collection

5 mL of ethylene diamine tetra acetic acid (EDTA) anticoagulation tubes (Invitrogen, CA, USA) were used to collect the peripheral blood from patients with HBV-ACLF and centrifuged for 10 minutes at 1000*g*, within two hours since acquisition. The plasma was stored at −80°C in aliquots before analysis. Plasma S100A8 and S100A9 were detected by human S100A8 ELISA Kit and human S100A9 ELISA Kit (Cusabio, Wuhan, China), respectively. Liver tissues were taken from 32 patients undergoing the transplantation for HBV-ACLF, while normal liver tissue was obtained from 19 patients who underwent liver hemangioma surgery. Liver tissue samples of 10 CHB patients were obtained by needle biopsy. Quickly freeze and store the tissues at −80°C as soon as possible after obtaining it. Clinical data about laboratory findings (including biochemical markers, alpha-fetoprotein, routine blood analyses, coagulative function, and HBV index), complications, causative factors, organ failure events, history of chronic disease, and the information on the treatment received were collected in the hospital's work system. We calculated the following severity scores: model of end-stage liver disease scores (MELDs) [18], CLIF-Consortium ACLF scores (CLIF-C ACLFs), CLIF-Consortium Organ Failure scores (CLIF-C OFs) [19], Chronic Liver Failure-Sequential Organ Failure Assessment scores (CLIF-SOFAs) [1], Chinese Group on the Study of Severe Hepatitis B-ACLF scores (COSSH-ACLFs), and COSSH-ACLF II score [20]. Survival data from ACLF patients at day 28 and day 90 were collected by contacting patients and their family members or from medical records.

### 2.3. Definitions

In line with the APASL criteria, ACLF was outlined as that patient with antecedently diagnosed or unknown chronic liver disease/cirrhosis suffer from acute liver injury within four weeks, with serum total bilirubin (TB) level ≥5 mg/dL and international normalized ratio (INR) ≥1.5 (or a prothrombin activity greater than 40%) come with clinical ascites and/or hepatic encephalopathy (HE) [16]. Ascites assessment and grading were in accordance with the following criteria: Grade 1 ascites: mild ascites detectable only by ultrasonography, Grade 2 ascites: moderate symmetric abdominal distention, and Grade 3 ascites: massive ascites accompanied by significant abdominal distension [21]. HE was defined based on the West Haven criteria [22]. Diagnosis of cirrhosis was in the main supported results of previous liver biopsy, clinical presentation of biochemical parameters, previous decompensation, imaging proof of liver nodularity, and/or portal hypertension [2]. The diagnosed and classified infection include (i) spontaneous bacterial peritonitis: the count of ascitic fluid polymorphonuclear cell >250/mm^3^, (ii) urinary tract infection: white blood cell (WBC) in urine >10/high-power fields with urinary irritation symptoms and positive urine culture, (iii) pneumonia: radiological evidence of consolidation and a minimum of 2 of the subsequent criteria: signs of consolidation on physical examination, body temperature  <35°C or  >38°C, chest pain, cough and sputum, or dyspnea, and (iv) other bacterial infections, such as osteoarticular infection, catheter-related infection, and bacteriemia of unknown cause [23]. The COSSH-ACLF grades and European Association for the Study of the Liver-ACLF (EASL-ACLF) grades were defined based on the criteria outlined in previous reports [1, 2]. If the patients meet the inclusion criteria but do not reach the standard of ACLF grade 1, they are classified as ACLF Grade 0. Defined of organ failure: kidney failure (serum creatinine ≥2 mg/dL), liver failure (TB ≥ 12 mg/dL), circulation failure (treatment with a vasoactive agent), brain failure (grade III–IV HE), coagulation failure (INR ≥2.5 or thrombocyte count ≤20 × 10^9^/L), and lung failure (SpO_2_/FiO_2_ ≤214 or PaO_2_/FiO_2_ ≤200) [1]. The clinical course was estimated by comparison CLIF-SOFAs at admission and the last available measure of the score before death or discharge from the hospital, or within 28 days of diagnosis. Deterioration and improvement were defined as at least 2-point decrease or increase in the CLIF-SOFAs, respectively, while the steady course was defined as the difference between −1 and 1.

### 2.4. ACLF Treatment

All of the patients received standard medical treatment [16, 24], such as nutritional supplementation, antiviral therapy, absolute bed rest, ascites puncture, albumin infusion, and appropriate treatment for complications such as HE, gastrointestinal bleeding (GB), and hepatorenal syndrome (HRS). In addition, patients with bacterial infection (BI) were treated with antibiotics and subsequently adjusted according to the results of culture and antibiotic sensitivity tests. After comprehensive evaluation of the patient's clinical status, artificial liver support (ALS) was used for suitable patients.

### 2.5. Real-Time PCR

The liver tissues were homogenized for real-time PCR analysis. Total RNA was extracted with TRIzol (Invitrogen, CA, USA) after the homogenization of liver tissues and was reverse-transcribed to complementary DNA (cDNA) employing a PrimerScript RT Reagent Kit (Takara, Dalian, China). Quantification of mRNA levels was performed by real-time PCR. The relative S100A8 and S100A9 mRNA expression levels were estimated by the 2^−ΔΔCT^method and normalized to *β*-actin mRNA. The primer pairs used in the real-time PCR were as follows: S100A8, forward: 5′-ATGCCGTCTACAGGGATGACCT-3′, reverse: 5′-AGAATGAGGAACTCCTGGAAGTTA-3′; S100A9, forward: 5′-CTGAGCTTCGAGGAGTTCATCA-3′, reverse: 5′-CGTCACCCTCGTGCATCTTC-3′; and *β*-actin, forward: 5′-CACCATTGGCAATGAGCGGTTC-3′, reverse: 5′-AGGTCTTTGCGGATGTCCACGT-3′.

### 2.6. Statistical Analysis

SPSS software for Windows (IBM, NY, USA; version 26.0) was used for statistical analyses. Medians (IQR) were used for express the results of continuous variables and proportion (%) for categorical variables. Wilcoxon's nonparametric test or Student's *t*-test were used to evaluate the differences between the two groups of normally distributed data, and for the categorical variables, Fisher's exact test or chi-square test were used. An analysis of correlation was performed through Spearman's rank tests. Univariate and multivariate hazard ratios (HRs) for factors related to 28/90-day mortality were assessed by the Cox proportional hazard model. To identify independent predictors, multivariate analysis includes all factors with *P* < 0.05 in the univariate. The area under the receiver operating characteristic curve (AUROC) was chosen to compare the prognosticative values of various prognostic factors. Youden index (sensitivity + specificity −1) was calculated to pick up the optimal cutoff values, and the maximum value was served as the optimal cutoff points. The Kaplan–Meier method was chosen to analyze the survival rate at 28 and 90 days. All statistical tests were two-sided, and significance was described as *P* < 0.05.

## 3. Results

### 3.1. Patients and Baseline Characteristics

Table 1 shows the demographic characteristics of transplant-free patients supported their survival status at 90 days. A total of 42% (*n* = 79) of patients died within 90 days. Compared with survivors, nonsurvivors were older and they had higher TB, WBC, creatinine, INR, and prothrombin time (PT), while survivors had higher estimated glomerular filtration rate (eGFR) and platelet count. HBV reactivation was the foremost common causative event (45.5%), followed by bacterial infection (10.2%), alcoholism (7.0%), and the use of hepatotoxic drugs (5.3%). In addition, more complications and a higher proportion of organ failure were found in patients died within 90 days. All prognostic scores, including MELD, CLIF-C OF, CLIF-SOFA, COSSH-ACLF, CLIF-C ACLF, and COSSH-ACLF II scores, were higher in nonsurvivors when put next with the survivors (*P* < 0.001) (Table 1).

### 3.2. Plasma S100A8 and S100A9 Levels in HBV-ACLF Patients and Their Relationship with ACLF Grade

**Figure 1:**
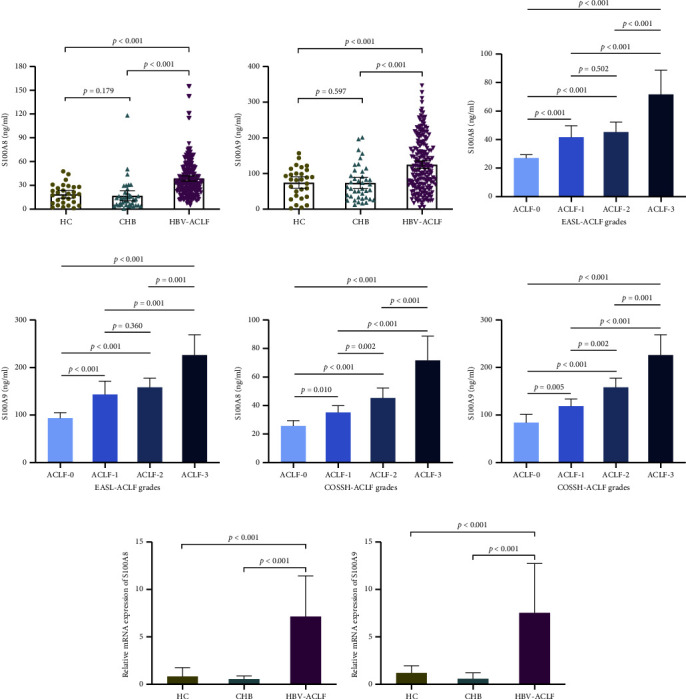
Baseline levels of plasma S100A8 and S100A9 in HBV-ACLF patients. (a) Comparison of plasma S100A8 (b) and S100A9 between HBV-ACLF patients, CHB patients, and HCs. (c) Comparison of plasma S100A8 (d) and S100A9 among different EASL-ACLF grade subgroups of patients (e) Comparison of plasma S100A8 and (f) S100A9 among different COSSH-ACLF grade subgroups of patients. (g) Comparison of S100A8 (h) and S100A9 mRNA levels between HBV-ACLF patients, CHB patients, and HCs. Horizontal lines and error bars represent media ±95% confidence interval (CI).

The baseline plasma levels of S100A8 and S100A9 were significantly higher in HBV-ACLF patients than in healthy controls (HCs) and CHB patients, while there was no distinction between HCs and CHB patients (Figures 1(a) and 1(b)). Moreover, plasma levels of S100A8 and S100A9 were also analyzed within 32 liver transplant patients. Compared with transplant-free patients, no vital distinction was discovered in plasma levels of S100A8 (*P* = 0.143) and S100A9 (*P* = 0.198) among patients with liver transplantation (Figure S1). Patients were graded supported the COSSH and EASL-ACLF criteria to assess the levels of S100A8 and S100A9. According to the EASL-ACLF criteria, there were 91 patients at grade 0, 36 were ACLF at grade 1, 45 were ACLF at grade 2, and 15 were ACLF at grade 3. Higher plasma S100A8 and S100A9 were found in ACLF-3 patients compared to ACLF-2/-1/-0 patients (Figures 1(c) and 1(d)). In accordance with the COSSH-ACLF criteria, there were 32 ACLF patients at grade 0, 95 were ACLF at grade 1, 45 were ACLF at grade 2, and 15 were ACLF at grade 3. Similarly, the plasma levels of S100A8 and S100A9 elevated gradually with the increase of ACLF grade (Figures 1(e) and 1(f)), denoting that elevation of S100A8 and S100A9 was related to a high ACLF grade.

In addition, mRNA expression levels of S100A8 and S100A9 were analyzed in liver tissue samples from 32 HBV-ACLF patients, 19 hepatic hemangioma patients (used as healthy controls), and 10 CHB patients. The result showed that relative mRNA expression levels of S100A8 and S100A9 were significantly higher in HBV-ACLF patients than in HCs and CHB patients (*P* < 0.001) (Figures 1(g) and 1(h).)

### 3.3. Association of S100A8 and S100A9 with Prognosis in HBV-ACLF Patients

**Figure 2:**
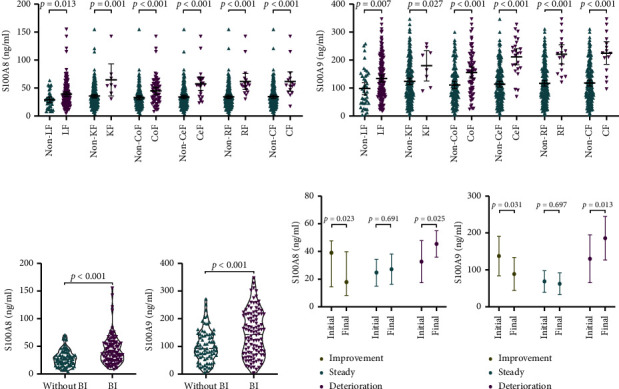
Association of plasma S100A8 and S100A9 levels with HBV-ACLF organ failure and disease progression. (a) Plasma S100A8 (b) and S100A9 distribution of HBV-ACLF patients with or without liver failure (LF), coagulation failure (CoF), kidney failure (KF), cerebral failure (CeF), respiratory failure (RF), and circulation failure (CF). (c) Comparison of plasma S100A8 and S100A9 between patients with or without BI. (d) Dynamic changes in plasma S100A8 and S100A9 between the initial (admission) and final (before death or discharge from the hospital or 28 days of diagnosis). Horizontal lines and error bars represent media ±95% confidence interval (CI).

We examined the association between plasma S100A8/S100A9 and complications in HBV-ACLF patients. Patients with kidney, liver, cerebral, coagulation, circulation, and respiratory failure (Figures 2(a) and 2(b)) had elevated plasma S100A8 and S100A9 than those without these conditions, as well as those with bacterial infections (Figure 2(c)). 27 patients with available follow-up serum samples were assessed to observe the dynamics of S100A8 and S100A9 levels during the patients' hospitalization. Compared with S100A8 and S100A9 levels at admission, levels of these two proteins at the final follow-up evaluation (before death or discharge, or 28 days after admission) were considerably increased in the deterioration group (*P*=0.025 and *P*=0.013, respectively), significantly decreased in the improvement group (*P*=0.023 and *P*=0.031, respectively), and were unchanged in the steady group (*P*=0.691 and *P*=0.697, respectively) (Figure 2(d)). However, since the sample size that used to analyze the dynamics of S100A8 and S100A9 levels was small, a larger sample size needs to be collected in the future to confirm whether plasma S100A8 and S100A9 closely connected with the clinical course of HBV-ACLF.

**Figure 3:**
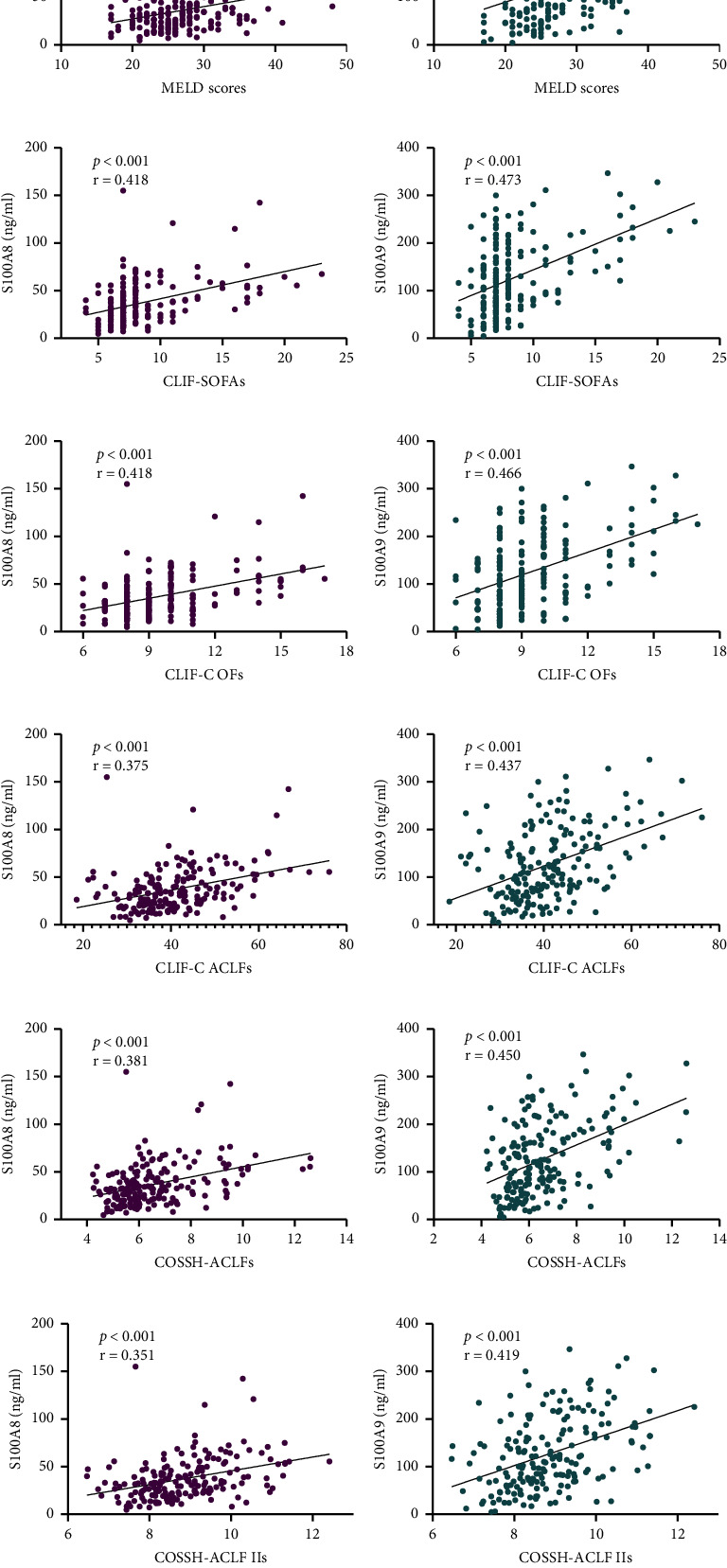
The correlations between the plasma S100A8 or S100A9 levels and prognostic scoring systems. (a) Spearman's correlation analyses between S100A8 and MELDs, (c) CLIF-SOFAs, (e) CLIF-C OFs, (g) CLIF-C ACLFs, (i) COSSH-ACLFs, and (k) COSSH-ACLF IIs. (b) Spearman's correlation analyses between S100A9 and MELDs, (d) CLIF-SOFAs, (f) CLIF-C OFs, (h) CLIF-C ACLFs, (j) COSSH-ACLFs, and (l) COSSH-ACLF IIs.

Additionally, Spearman rank correlation was used to estimate the correlations between plasma S100A8 or S100A9 levels and prognostic scoring systems. The results showed that plasma S100A8 and S100A9 at admission were positively connected with the MELDs, CLIF-C OFs, CLIF-SOFAs, COSSH-ACLFs, CLIF-C ACLFs, and COSSH-ACLF IIs (Figure 3). Similarly, the relative mRNA expression levels of S100A8 and S100A9 in liver tissues of HBV-ACLF patients showed a strong relationship with the prognostic scoring systems mentioned above (Figure S2).

### 3.4. Predictors of 28-Day and 90-Day Transplant-free Mortality Risk

Furthermore, we used the univariate and multivariate Cox proportional hazards analysis to discover the predictors of 28/90-day mortality in HBV-ACLF patients. In univariate analysis, age, WBC count, creatinine, INR, ascites grade, platelet (PLT) count, TB, HE grade, BI, S100A8, and S100A9 were connected with 28/90-day mortality. In multivariate analyses, the baseline age (HR: 1.006; 95% confidence interval (CI): 1.002–1.011; and *P*=0.009), creatinine (HR: 1.005; 95% CI: 1.001–1.010; and *P*=0.019), INR (HR: 1.620; 95% CI: 1.227–2.138; and *P*=0.001), HE grade (HR: 1.330; 95% CI: 1.023–1.731; and *P*=0.034), S100A8 (HR: 1.027; 95% CI: 1.007–1.048; and *P*=0.026), and S100A9 (HR: 1.009; 95% CI: 1.001–1.017; and *P*=0.007) were significant independent predictors of 28-day mortality (Table 2). Moreover, age (HR: 1.034; 95% CI: 1.007–1.061; and *P*=0.013), TB (HR: 1.002; 95% CI: 1.001–1.003; and *P*=0.002), HE grade (HR: 1.499; 95% CI: 1.173–1.916; and *P*=0.001), INR (HR: 2.362; 95% CI: 1.740–3.207; and *P* < 0.001), S100A8 (HR: 1.023; 95% CI: 1.011–1.035; and *P*=0.004), and S100A9 (HR: 1.008; 95% CI: 1.004–1.012; and *P* < 0.001) were significant independent predictors of 90-day mortality (Table 2).

**Figure 4:**
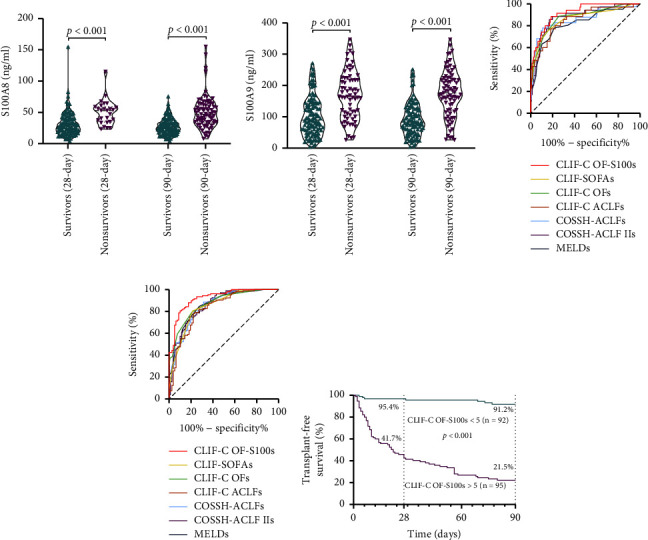
Prognostic performance of the ACLF prognostic scoring systems. (a) Plasma S100A8 (b) and S100A9 distribution of HBV-ACLF patients survive or no survive at 28 or 90 days. (c) ROC curves of prognostic models in predicting 28-day mortality (d) and 90-day mortality in HBV-ACLF patients. (e) Probability of transplant-free survival at 28/90-day on the basis of the CLIF-C OF-S100s cutoff value in ACLF patients. The log-rank test was not to compare the Kaplan–Meier curves. Horizontal lines and error bars represent media ±95% confidence interval (CI).

As a classical model, CLIF-C OFs is extensively used in ACLF prognosis prediction. The identified predictors of 90-day mortality, such as TB, INR, and HE grade, can be used to assess organ failure, and they are all components of the CLIF-C OFs. Moreover, patients who died within 28 or 90 days would demonstrate higher S100A8 and S100A9 than survivors (*P* < 0.001) (Figure 4(a) and 4(b)). Therefore, to improve the prognostic value of the CLIF-C OFs, we developed the CLIF-C OF-S100s (0.435 × CLIF-C OFs + 0.013 × S100A8 + 0.006 × S100A9) using the composite marker (S100A8 plus S100A9) in conjunction with CLIF-C OFs in accordance with the CLIF-C OFs controlled multivariate analysis (Table S1). The prognostic values of the CLIF-C OF-S100s and other prognostic scoring systems were further evaluated by receiver operating characteristic (ROC) analysis, and their areas under curve (AUC) were calculated. In predicting the 28-day mortality, the AUC for the CLIF-C OF-S100s was 0.918 (95% CI, 0.873–0.964), which was higher than that for the MELDs, CLIF-C OFs, CLIF-SOFAs, COSSH-ACLFs, CLIF-C ACLFs, and COSSH-ACLF IIs (Figure 4(c) and Table S2). The AUC of the CLIF-C OF-S100s for predicting 90-day mortality was 0.923 (95% CI, 0.887–0.961), which was superior to other scoring systems (Figure 4(d) and Table S2).

In addition, for the CLIF-C OF-S100s, the optimal cutoff value in predicting 90-day mortality risk was 5, which providing a sensitivity of 89.9% and specificity of 80.6%. The Kaplan–Meier survival analyses found that patients with a low CLIF-C OF-S100s (<5) had lower mortality at 28 and 90 days than those with CLIF-C OF-S100s > 5 (28-day survival rate: 95.4% vs. 41.7%; 90-day survival rate: 91.2% vs. 21.5%, *P* < 0.001) (Figure 4(e)).

## 4. Discussion

We investigated the usefulness of plasma S100A8 and S100A9 for the prediction of progression and prognosis in HBV-ACLF patients during this study. We found that both proteins could be used as prognostic biomarkers not only as they reflected organ injury but also as they were considerably related to 28/90-day mortality. Similarly, compared with CHB patients and normal liver, higher hepatic S100A8 and S100A9 mRNA levels were demonstrated in HBV-ACLF patients. In addition, plasma S100A8 and S100A9 were combined with the CLIF-C OFs, and the CLIF-C OF-S100s demonstrated the best prognostic utility compared with all other prognostic scoring systems.

In the development of ACLF, necroinflammation is a key pathophysiological process [25, 26]. Necroptotic cells contributed to the release of damage-associated molecular pattern molecules (DAMPs), including S100A8 and S100A9, which can initiate inflammatory pathways to secrete proinflammatory cytokines, and thereby lead to hepatocyte apoptosis and necrosis [27, 28]. As warning/danger signals for the host, the levels of S100A8 and S100A9 increase in diverse inflammatory diseases [13, 29, 30], which is in keeping with our results that showed that compared with CHB patients and HCs, the expression levels of S100A8 and S100A9 in plasma and hepatic were significantly higher in HBV-ACLF patients. S100A8 and S100A9 can be combined to form a stable heterodimer (S100A8/A9), which was found to be a more sensitive biomarker in the treatment response and inflammatory activity compared with routine inflammation indexes [31, 32]. Before releasing into the bloodstream, S100A8 and S100A9 are local production and secretion at the site of inflammatory, and therefore, they have the potential to act as the host's first response to inflammatory conditions and may have a decisive kinetic advantage [8]. Therefore, S100A8, S100A9, and S100A8/A9 complex are perhaps more appealing as biomarkers than the commonly used inflammatory biomarkers. Likewise, therapies targeting these proteins could also be more advantageous in inflammation-associated diseases. Experimental studies have shown that through the inhibition of the necroptosis-S100A9-necroinflammation axis, M2-like macrophages can exert hepatoprotection in ACLF [27]. S100A8 induces increased phosphorylation of interleukin-1 receptor-associated kinase 1 (IRAK-1), translocation of myeloid differentiation factor 88 (MyD88), and activation of nuclear factor kappa-B (NF-*κ*B) in septic shock, thereby resulting in increased of tumor necrosis factor-alpha (TNF-*α*) in phagocytes, and the absence of S100A8 and S100A9 protects mice from lipopolysaccharides-induced lethal shock [33]. Therefore, these two proteins may also be potential therapeutic targets in systemic inflammatory response disease.

In addition, we investigated the correlations of plasma S100A8/S100A9 levels and other prognostic scoring systems to explore the latent of S100A8 and S100A9 to be used as biomarkers for HBV-ACLF. The results found that the two proteins had positive correlation between all of the scoring systems, and the same results were obtained for the relative mRNA levels of S100A8 and S100A9 in liver tissue. The above results recommend that S100A8 and S100A9 directly reflect the severity of HBV-ACLF.

Another main aspect of concern in the study was that high S100A8 and S100A9 levels in HBV-ACLF patients were related to mortality, and both proteins were independent predictors of 28/90-day mortality. Considering the dismal short-term prognosis of ACLF, there is an urgent need for accurate prognostic score to aid in liver transplantation decisions. To date, several scores have been reported on predicting the mortality in patients with ACLF [1, 18–20]. A previous study explored the prophetical ability of some traditional scores and found that according to the APASL criteria, the MELDs model was the only one that was significantly connected with the mortality in ACLF patients. However, in keeping with the EASL/American Association for the Study of Liver Diseases (AASLD) criteria, none of the three scores including MELD, Child–Pugh, and albumin-bilirubin (ALBI) were considerably related to the in-hospital mortality [34]. Another study found that compared with other scores, CLIF-C ACLFs had the highest AUROC in predicting 28-day mortality [35], while other studies reported that the predictive accuracy of CLIF-SOFAs and the simpler CLIF-C OFs for mortality were considerably higher than that of CLIF-C ACLFs and MELDs [36, 37]. The CLIF-C OFs is a model that quantifies severity of organ dysfunction and calculates the sum to estimate the prognosis of ACLF. Apart from reflecting multiorgan dysfunction of HBV-ACLF patients, S100A8 and S100A9 may also reflect the systemic inflammatory milieu occurring in several inflammatory diseases [9, 27, 29], which is not captured by the CLIF-C OFs variables. Our new prognostic model combined S100A8 and S100A9 levels with the CLIF-C OFs, resulting in improved accuracy of predicting mortality in HBV-ACLF patients. Nevertheless, there have been some limitations to the study. First, we enrolled only HBV-ACLF patients, and whether these results could be used in patients with other causes of ACLF still needs further validation. Second, the prognostic efficacy of S100A8, S100A9, and CLIF-C OF-S100s should be confirmed in a multicenter, large sample size study.

## 5. Conclusion

In conclusion, S100A8 and S100A9 are independent predictors of short-run mortality and promising biomarkers within the analysis of prognosis and risk stratification in HBV-ACLF patients. Furthermore, the composite score that combines S100A8 and S100A9 with the CLIF-C OFs significantly improves the accuracy of prognosis prediction in patients with HBV-ACLF.

## Figures and Tables

**Table 1:** Baseline characteristics of patients with HBV-ACLF.

	Total	Survivors	Nonsurvivors	*P* Value
Total (*n*)	187	108	79	
Male	164 (87.7%)	97 (89.8%)	67 (84.8%)	0.303
Age (years)	46.0 (38.0–53.0)	43.0 (36.0–51.0)	50.0 (44.0–57.0)	<0.001
ALT (U/L)	103.5 (59.3–258.8)	97.0 (59.5–207.5)	117.0 (62.0–290.0)	0.985
AST (U/L)	110.0 (70.0–239.0)	105.5 (67.0–205.5)	112.0 (74.0–258.5)	0.315
ALB (g/L)	35.0 (31.9–38.0)	35.0 (31.0–38.0)	35.0 (32.0–38.0)	0.527
TB (umol/L)	336.8 (219.4–485.5)	274.2 (170.1–428.7)	436.5 (298.2–569.0)	<0.001
WBC (^*∗*^10^^9^/L)	6.4 (4.2–8.4)	6.0 (4.1–7.5)	7.4 (4.8–9.7)	0.013
HB (g/L)	109.0 (97.0–126.0)	109.5 (99.0–127.5)	108.0 (95.0–124.0)	0.905
PLT (*∗*10^^9^/L)	71.0 (47.3–113.8)	82.0 (56.0–135.0)	56.0 (42.3–82.5)	<0.001
Creatinine (umol/L)	69.0 (57.0–86.5)	67.0 (56.0–77.0)	75.0 (58.5–98.1)	0.003
eGRF (mL/min)	111.1 (84.0–140.3)	116.5 (96.2–142.1)	96.9 (70.7–137.0)	0.007
AFP (ug/L)	44.5 (11.3–169.7)	62.1 (18.4–219.5)	26.0 (6.1–101.8)	0.177
INR	2.2 (1.8–2.7)	1.9 (1.7–2.3)	2.7 (2.3–3.5)	<0.001
PT (s)	23.7 (19.8–28.9)	20.8 (18.2–24.8)	28.2 (24.0–35.9)	<0.001

*Precipitating event, N (%)*				
HBV reactivation	85 (45.5%)	55 (50.9%)	30 (38.0%)	0.079
Superimposed HAV or HEV	3 (1.6%)	2 (1.9%)	1 (1.3%)	0.759
Bacterial infection	19 (10.2%)	10 (9.3%)	9 (11.4%)	0.633
Drug use	10 (5.3%)	4 (3.7%)	6 (7.6%)	0.243
Alcoholism	13 (7.0%)	8 (7.4%)	5 (6.3%)	0.775
Others	57 (30.5%)	29 (26.9%)	28 (35.4%)	0.207

*Underlying liver disease, N (%)*				
Chronic hepatitis B	98 (52.4%)	62 (57.4%)	36 (45.6%)	0.109
Compensated cirrhosis	50 (26.7%)	27 (25.0%)	23 (29.1%)	0.530
Decompensated cirrhosis	39 (20.9%)	19 (17.6%)	20 (25.3%)	0.156

*Complications, N (%)*				
Ascites	113 (60.4%)	57 (52.8%)	56 (70.9%)	0.012
Gastrointestinal hemorrhage	12 (6.4%)	2 (1.9%)	10 (12.7%)	0.003
Hepatic encephalopathy	61 (32.6%)	16 (14.8%)	45 (57.0%)	<0.001
Bacterial infection	108 (57.8%)	52 (48.1%)	56 (70.9%)	0.002

*Organ failure, N (%)*				
Liver	147 (78.6%)	74 (68.5%)	73 (92.4%)	<0.001
Kidney	8 (4.3%)	2 (1.9%)	6 (7.6%)	0.055
Coagulation	64 (34.2%)	17 (15.7%)	47 (59.5%)	<0.001
Lung	17 (9.1%)	0 (0%)	17 (21.5%)	<0.001
Cerebral	24 (12.8%)	1 (0.9%)	23 (29.1%)	<0.001
Circulation	15 (8.0%)	0 (0%)	15 (19.0%)	<0.001

*Prognostic score*				
CLIF-SOFAs	7.0 (7.0–9.0)	7.0 (6.0–7.0)	9.0 (8.0–13.0)	<0.001
CLIF-C OFs	9.0 (8.0–10.0)	8.0 (8.0–9.0)	10.0 (10.0–13.0)	<0.001
CLIF-C ACLFs	40.0 (34.5–46.1)	36.3 (32.8–41.1)	46.1 (42.4–52.7)	<0.001
COSSH-ACLFs	6.2 (5.5–7.2)	5.7 (5.2–6.2)	7.4 (6.5–9.2)	<0.001
COSSH-ACLF IIs	8.7 (8.1–9.5)	8.3 (7.8–8.8)	9.6 (9.1–10.3)	<0.001
MELDs	27.0 (23.5–30.8)	24.0 (22.0–27.0)	30.0 (28.0–34.8)	<0.001

Values are expressed as median (IQR) or number of patients (%). HBV-ACLF: hepatitis B virus-related acute-on-chronic live failure; ALT: alanine aminotransferase; AST: aspartate transaminase; ALB: albumin; TB: total bilirubin; WBC: white blood cell; HB: Hemoglobin; PLT: blood platelet; eGFR: estimated glomerular filtration rate; AFP: alpha-fetoprotein; INR: international normalized ratio; PT: prothrombin time; HBV: hepatitis B virus; HAV: hepatitis A virus; HEV: hepatitis E virus; CLIF-SOFAs: Chronic Liver Failure-Sequential Organ Failure Assessment score; CLIF-C OFs: CLIF-Consortium Organ Failure score; CLIF-C ACLFs: CLIF-Consortium ACLF score; COSSH-ACLFs: Chinese Group on the Study of Severe Hepatitis B-ACLF score; and MELDs: Model of End-Stage Liver Disease score.

**Table 2:** Predictors of 28-day and 90-day mortality in HBV-ACLF patients.

Predictors (28-day mortality)	Univariate analysis		Multivariate analysis	
	HR (95% CI)	*P* value	HR (95% CI)	*P* value
Age	1.038 (1.016–1.060)	0.001	1.006 (1.002–1.011)	0.009
Male sex	0.749 (0.436–1.286)	0.295		
WBC (^*∗*^10^9/L)	1.074 (1.014–1.138)	0.015	1.041 (0.996–1.123)	0.294
HB (g/L)	1.000 (0.990–1.010)	0.966		
PLT (^*∗*^10^9/L)	0.989 (0.983–0.996)	0.001	0.996 (0.988–1.003)	0.230
ALB (g/L)	0.984 (0.915–1.059)	0.669		
TB (umol/L)	1.003 (1.002–1.004)	<0.001	1.002 (1.000–1.004)	0.089
Creatinine (umol/L)	1.006 (1.003–1.008)	<0.001	1.005 (1.001–1.010)	0.019
INR	2.222 (1.799–2.745)	<0.001	1.620 (1.227–2.138)	0.001
AFP (ug/L)	0.997 (0.993–1.001)	0.192		
Ascites grade	2.009 (1.362–2.964)	<0.001	0.912 (0.670–1.241)	0.558
HE grade	1.933 (1.598–2.338)	<0.001	1.330 (1.023–1.731)	0.034
Bacterial infection	1.225 (0.738–2.032)	0.008	0.641 (0.341–1.206)	0.168
S100A8 (ng/mL)	1.018 (1.011–1.025)	<0.001	1.027 (1.007–1.048)	0.026
S100A9 (ng/mL)	1.014 (1.009–1.019)	<0.001	1.009 (1.001–1.017)	0.007

*Predictors (90-day mortality)*				
Age	1.037 (1.017–1.058)	<0.001	1.034 (1.007–1.061)	0.013
Male sex	0.754 (0.410–1.390)	0.366		
WBC (∗10^9/L)	1.067 (1.011–1.127)	0.018	1.021 (0.933–1.118)	0.651
HB (g/L)	1.001 (0.991–1.010)	0.901		
PLT (∗10^9/L)	0.990 (0.984–0.996)	0.001	1.001 (0.994–1.007)	0.809
ALB (g/L)	0.999 (0.961–1.038)	0.947		
TB (umol/L)	1.003 (1.002–1.004)	<0.001	1.002 (1.001–1.003)	0.002
Creatinine (umol/L)	1.007 (1.004–1.010)	<0.001	0.998 (0.995–1.002)	0.304
INR	2.804 (2.208–3.561)	<0.001	2.362 (1.740–3.207)	<0.001
AFP (ug/L)	0.999 (0.997–1.001)	0.225		
Ascites grade	1.591 (1.262–2.006)	<0.001	1.183 (0.905–1.547)	0.220
HE grade	1.914 (1.606–2.281)	<0.001	1.499 (1.173–1.916)	0.001
Bacterial infection	2.132 (1.277–3.557)	0.004	1.122 (0.605–2.083)	0.715
S100A8 (ng/mL)	1.023 (1.017–1.029)	<0.001	1.023 (1.011–1.035)	0.004
S100A9 (ng/mL)	1.012 (1.009–1.015)	<0.001	1.008 (1.004–1.012)	<0.001

CI: confidence interval; HR: hazard ratio; WBC: white blood cell; HB: Hemoglobin; PLT: blood platelet; ALB: albumin; TB: total bilirubin; INR: international normalized ratio; AFP: alpha-fetoprotein; and HE: Hepatic encephalopathy.

## Data Availability

The data used to support the findings of the study are fully available upon request.

## Supplementary Materials

Supplementary Materials Table S1: risk factors associated with transplant-free 90-day mortality in patients with HBV-ACLF according to a multivariate Cox proportional hazards model. Table S2: comparison of receiver operating characteristic curves for different prognostic models in predicting 28-day and 90-day mortality in HBV-ACLF patients. Figure S1: (a) comparison of plasma S100A8 and (b) S100A9 between HBV-ACLF patients with or without liver transplantation. Horizontal lines and error bars represent media ± 95% confidence interval (CI). Figure S2: the correlations between the relative mRNA expression of S100A8 or S100A9 and prognostic scoring systems. (a) Spearman's correlation analyses between relative mRNA expression of S100A8 and MELDs, (c) CLIF-SOFAs, (e) CLIF-C OFs, (g) CLIF-C ACLFs, (i) COSSH-ACLFs, and (k) COSSH-ACLF IIs. (b) Spearman's correlation analyses between relative mRNA expression of S100A9 and MELDs, (d) CLIF-SOFAs, (f) CLIF-C OFs, (h) CLIF-C ACLFs, (j) COSSH-ACLFs, and (l) COSSH-ACLF IIs.
