# Supplementary material for: Plasma S100A8 and S100A9 Are Strong Prognostic Factors for Hepatitis B Virus-Related Acute-on-Chronic Liver Failure
Source: Can J Gastroenterol Hepatol. 2023 Jul 10;2023:6164611. doi: 10.1155/2023/6164611 (PMC10352535; doi:10.1155/2023/6164611)
Supplement: Supplementary Materials — Table S1: risk factors associated with transplant-free 90-day mortality in patients with HBV-ACLF according to a multivariate Cox proportional hazards model. Table S2: comparison of receiver operating characteristic curves for different prognostic models in predicting 28-day and 90-day mortality in HBV-ACLF patients. Figure S1: (a) comparison of plasma S100A8 and (b) S100A9 between HBV-ACLF patients with or without liver transplantation. Horizontal lines and error bars represent media ± 95% confidence interval (CI). Figure S2: the correlations between the relative mRNA expression of S100A8 or S100A9 and prognostic scoring systems. (a) Spearman's correlation analyses between relative mRNA expression of S100A8 and MELDs, (c) CLIF-SOFAs, (e) CLIF-C OFs, (g) CLIF-C ACLFs, (i) COSSH-ACLFs, and (k) COSSH-ACLF IIs. (b) Spearman's correlation analyses between relative mRNA expression of S100A9 and MELDs, (d) CLIF-SOFAs, (f) CLIF-C OFs, (h) CLIF-C ACLFs, (j) COSSH-ACLFs, and (l) COSSH-ACLF IIs. [file 6164611.f1.docx]

**Plasma S100A8 and S100A9 are strong prognostic factors for hepatitis B virus–related acute-on-chronic liver failure**

**Supplementary Tables**

**Table S1.** Risk factors associated with transplant-free 90-day mortality in patients with HBV-ACLF according to a multivariate Cox proportional-hazards model

|  | Regression coefficient | HR (95% confidence interval) | *P* value |
| --- | --- | --- | --- |
| S100A8 | 0.013 | 1.014 (1.003-1.024) | 0.002 |
| S100A9 | 0.006 | 1.006 (1.002-1.010) | <0.001 |
| CLIF-C OFs | 0.435 | 1.546 (1.378-1.734) | <0.001 |

HBV-ACLF, hepatitis B virus–related acute-on-chronic live failure; HR, hazard ratio; CLIF-C OFs, CLIF-Consortium Organ Failure score.

**Table S2.** Comparison of receiver operating characteristic curves for different prognostic models in predicting 28-day and 90-day mortality in HBV-ACLF patients

|  | 28-day | | | |  | 90-day | | | |
| --- | --- | --- | --- | --- | --- | --- | --- | --- | --- |
|  | AUC | 95% CI | Sensitivity, % | Specificity, % |  | AUC | 95% CI | Sensitivity, % | Specificity, % |
| CLIF-C OF-S100s | 0.918 | (0.873-0.964) | 88.6 | 81.5 |  | 0.923 | (0.887-0.961) | 89.9 | 80.6 |
| CLIF-SOFAs | 0.862 | (0.786-0.939) | 75.0 | 84.8 |  | 0.848 | (0.792-0.904) | 78.5 | 80.6 |
| CLIF-C OFs | 0.875 | (0.806-0.943) | 88.9 | 74.2 |  | 0.872 | (0.817-0.927) | 79.0 | 79.6 |
| CLIF-C ACLFs | 0.855 | (0.782-0.928) | 77.1 | 81.6 |  | 0.829 | (0.767-0.890) | 77.9 | 78.1 |
| COSSH-ACLFs | 0.861 | (0.787-0.935) | 78.1 | 89.0 |  | 0.852 | (0.796-0.908) | 78.4 | 88.6 |
| COSSH-ACLF IIs | 0.880 | (0.816-0.944) | 88.6 | 80.1 |  | 0.861 | (0.807-0.915) | 81.0 | 75.0 |
| MELDs | 0.824 | (0.745-0.902) | 78.1 | 78.6 |  | 0.859 | (0.807-0.911) | 72.2 | 84.8 |

HBV-ACLF, hepatitis B virus–related acute-on-chronic live failure; CI, confidence interval; AUC, areas under curve; CLIF-SOFAs, Chronic Liver Failure-Sequential Organ Failure Assessment score; CLIF-C OFs, CLIF-Consortium Organ Failure score; CLIF-C OF-S100s, a novel prognostic score based on S100A8, S100A9, and CLIF-C OFs; CLIF-C ACLFs, CLIF-Consortium ACLF score; COSSH-ACLFs, Chinese Group on the Study of Severe Hepatitis B-ACLF score; MELDs, Model of end-stage liver disease score.

**Supplementary figure**

**
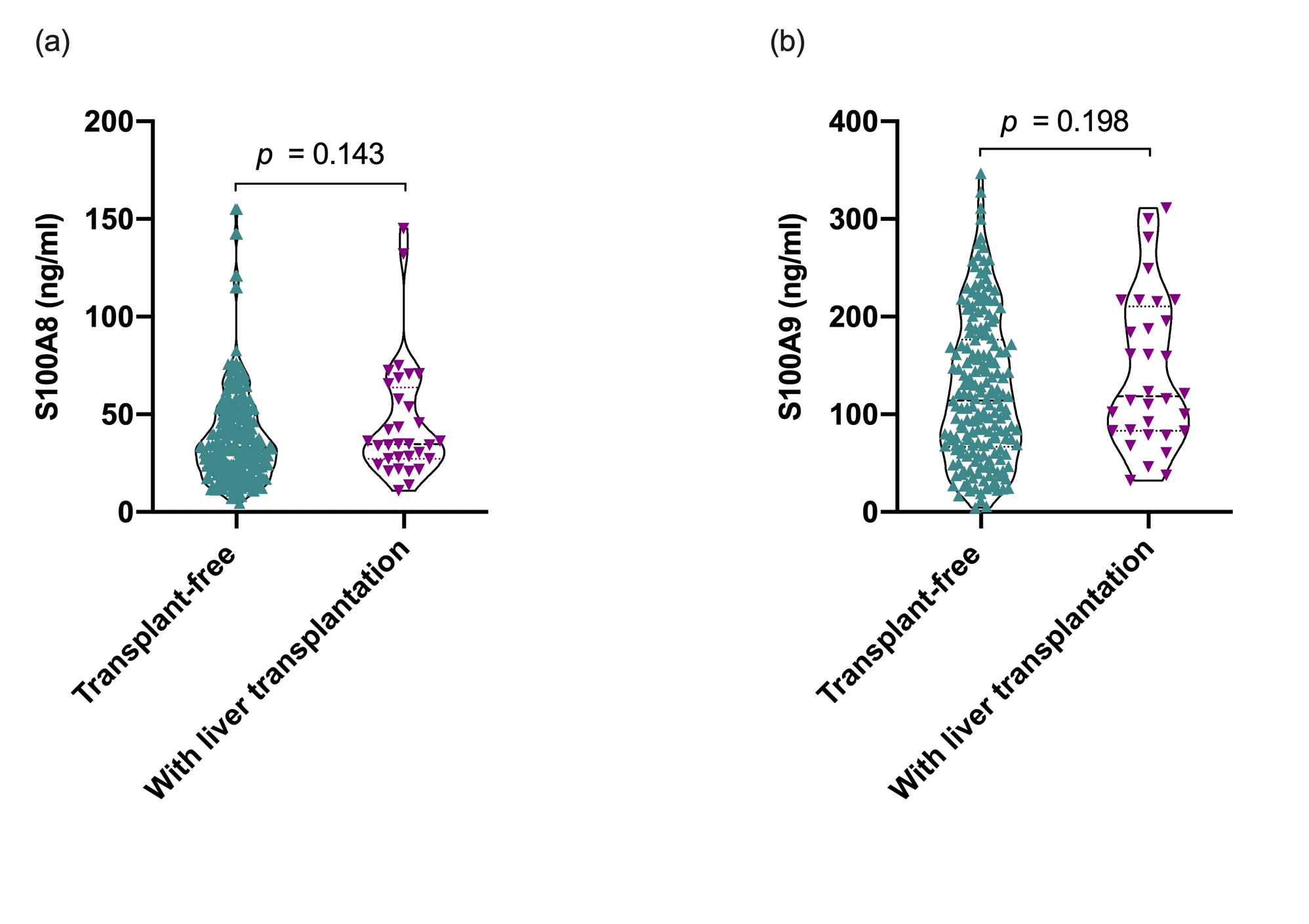
**

**Figure S1. (a) Comparison of plasma S100A8, (b) and S100A9 between HBV-ACLF patients with or without liver transplantation.** Horizontal lines and error bars represent media ± 95% confidence interval (CI).

**
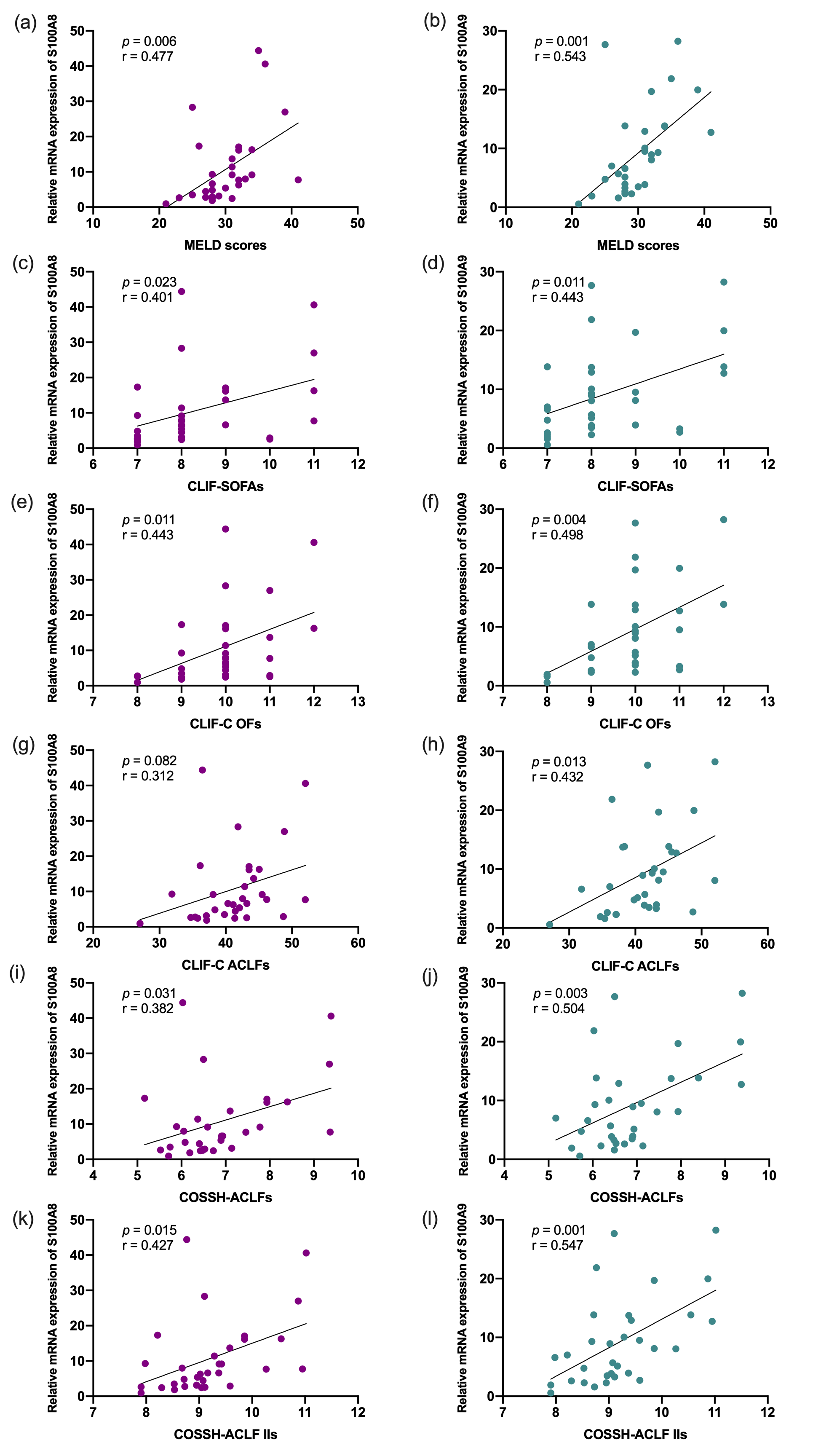
**

**Figure S2. The correlations between the relative mRNA expression of S100A8 or S100A9 and prognostic scoring systems.** (a) Spearman’s correlation analyses between relative mRNA expression of S100A8 and MELDs, (b) CLIF-SOFAs, (e) CLIF-C OFs, (g) CLIF-C ACLFs, (i) COSSH-ACLFs, (k) and COSSH-ACLF IIs. (b) Spearman’s correlation analyses between relative mRNA expression of S100A9 and MELDs, (d) CLIF-SOFAs, (f) CLIF-C OFs, (h) CLIF-C ACLFs, (j) COSSH-ACLFs, (l) and COSSH-ACLF IIs.
